# The Role of BDNF as a Biomarker in Cognitive and Sensory Neurodegeneration

**DOI:** 10.3390/jpm13040652

**Published:** 2023-04-10

**Authors:** Anna Pisani, Fabiola Paciello, Valeria Del Vecchio, Rita Malesci, Eugenio De Corso, Elena Cantone, Anna Rita Fetoni

**Affiliations:** 1 Department of Otolaryngology Head and Neck Surgery, Università Cattolica del Sacro Cuore, 00168 Rome, Italy; 2 Department of Neuroscience, Università Cattolica del Sacro Cuore, 00168 Rome, Italy; 3 Fondazione Policlinico Universitario A. Gemelli IRCCS, 00168 Rome, Italy; 4 Department of Neuroscience, Reproductive Sciences and Dentistry-Audiology Section, University of Naples Federico II, 80131 Naples, Italy; 5 Department of Neuroscience, Reproductive Sciences and Dentistry-ENT Section, University of Naples Federico II, 80131 Naples, Italy

**Keywords:** BDNF, biomarker, neurodegenerative disease, cochlea, hearing loss

## Abstract

Brain-derived neurotrophic factor (BDNF) has a crucial function in the central nervous system and in sensory structures including olfactory and auditory systems. Many studies have highlighted the protective effects of BDNF in the brain, showing how it can promote neuronal growth and survival and modulate synaptic plasticity. On the other hand, conflicting data about BDNF expression and functions in the cochlear and in olfactory structures have been reported. Several clinical and experimental research studies showed alterations in BDNF levels in neurodegenerative diseases affecting the central and peripheral nervous system, suggesting that BDNF can be a promising biomarker in most neurodegenerative conditions, including Alzheimer’s disease, shearing loss, or olfactory impairment. Here, we summarize current research concerning BDNF functions in brain and in sensory domains (olfaction and hearing), focusing on the effects of the BDNF/TrkB signalling pathway activation in both physiological and pathological conditions. Finally, we review significant studies highlighting the possibility to target BDNF as a biomarker in early diagnosis of sensory and cognitive neurodegeneration, opening new opportunities to develop effective therapeutic strategies aimed to counteract neurodegeneration.

## 1. Introduction

Neurotrophic factors include a large family of dimeric polypeptides such as nerve growth factor (NGF), brain-derived neurotrophic factor (BDNF), neurotrophin-3 (NT-3), and NT-4/5, known to play a crucial role in neuronal growth, differentiation, and survival as well as in regulation of neuronal structure and functions and not only in neurodevelopment but also in adult brain plasticity [1,2,3]. NGF, BDNF, and NT-3 functionality requires their binding to specific receptors, namely the tropomyosin receptor kinases (TrkA, TrkB, and TrkC), leading to the activation of different intracellular signalling pathways [4,5].

Among all neurotrophins, the alteration of BDNF, one of the most diffuse and extensively studied in the mammalian brain, has been proposed as a biomarker of several neurodegenerative conditions affecting both the peripheral and central nervous system (CNS) [6,7,8]. Indeed, the BDNF/TrkB signalling pathway is involved in brain development and neuronal plasticity as well as in inflammatory processes, pain, hearing development [9,10,11,12], neurogenesis, differentiation, survival, and synaptic plasticity [9,13,14].

TrkB has an extracellular domain with glycosylation sites, a transmembrane domain, and an intracellular tyrosine kinase domain. BDNF binding to the extracellular domain stimulates receptor dimerization and induces autophosphorylation of the intracellular tyrosine kinase domain. This step is crucial for the recruitment of several enzymes, namely phosphatidylinositol 3-kinase (PI3K), mitogen-activated protein kinase (MAPK), phospholipase C-γ (PLC-γ), and guanosine triphosphate hydrolases (GTP-ase) of the Ras homolog (Rho) gene family [15,16]. The recruitment of these signalling proteins is necessary for the activation of phosphatidylinositol 3-kinase (PI3K)/Akt, which can induce the deactivation of proapoptotic targets and the extracellular signal-regulated kinase (ERK), leading to the transcription of genes associated with neuronal survival, respectively [17,18].

Furthermore, BDNF exhibits a potent role in neuroprotection and/or brain regeneration upon several types of damages, promoting the resilience of neuronal cells against neurodegeneration [19,20].

Considering the double role of BDNF as a biomarker of neuronal plasticity and a protective factor against sensory and cognitive neurodegeneration, here, we will review the molecular mechanisms underlying BDNF effects, summarizing data supporting its role in the brain and in sensory systems such as the olfactory system (OS) and in the auditory peripheral organ, the cochlea. Finally, we summarize studies supporting the possibility to use BDNF as an early biomarker in sensory and cognitive neurodegenerations such as hearing loss and dementia.

## 2. The Antagonist Role of Pro-BDNF and mBDNF

Similarly to other neurotrophins, BDNF is initially produced as a precursor protein (pro-BDNF) located in neuronal dendrites or axons [21,22]. The pro-BDNF can be proteolytically cleaved by different proteases to generate mature BDNF (mBDNF), and both forms accumulate into vesicles from which they are secreted.

For many years, it has been assumed that only secreted mBDNF is a biologically active agent, whereas pro-BDNF is an inactive precursor exclusively located in the intracellular compartment. Nevertheless, several observations highlight the specific biological activity of pro-BDNF in CNS as an independent ligand [22,23,24]. Indeed, both pathological and physiological conditions can trigger pro-BDNF secretion [25]. Pang and collaborators reported that the extracellular proteinase plasmin is responsible for the transition of pro-BDNF, released in hippocampal synapses, to mBDNF, which is known to modulate synaptic plasticity [26]. Interestingly, many studies demonstrated that mBDNF and pro-BDNF interact with different receptors associated with opposed biological effects on cellular functions [27]. Pro-BDNF strongly binds p75^NTR^, a member of the tumour necrosis factor (TNF) receptor superfamily, which can induce long-term depression (LTD) and apoptosis mechanisms [27,28,29,30,31]. The mBDNF specifically binds TrkB [1,32,33] and exerts its protective effects by the activation of different signalling pathways, leading to cell survival and proliferation [30,34,35] and axon and dendrite growth and facilitating long-term potentiation (LTP) with the improvement of dendritic spine maturation [36,37,38]. The co-expression of both TrkB and p75^NTR^ receptors leads to an increase in mBDNF binding specificity [39]. In physiological conditions, the pro-BDNF/mBDNF balance is finely regulated in the CNS [40]. Nevertheless, considering that pro-BDNF has opposing biological effects compared to its mature form, this proteolytic process can be considered a potential tool to promote BDNF maturation.

BDNF expression is regulated during transcription by different promoters that control activity-dependent and tissue-specific expression [41,42] by translation and by post-translational modifications. There are various mechanisms generating multiple transcripts and protein isoforms, and many of these are of unrevealed physiological function. Each transcript is modulated by several endogenous and exogenous factors such as neuronal activity [43], exercise [44], stress [45], and antidepressants [46,47] and hormones such as oestrogens [48,49]. Moreover, epigenetic mechanisms can affect the expression of specific BDNF exons such as DNA methylation [50,51], suggesting that mBDNF levels can be dynamically influenced by environmental factors. During development, BDNF is involved in neuronal survival, growth, and differentiation in several brain regions including the hippocampus, cerebellum, and cortex [1]. Multiple pieces of evidence highlight the central role of BDNF in the formation of new neurons, a process known as neurogenesis [52,53,54,55], but also in the establishment and maintenance of synapses between neurons [56,57,58].

A mechanism involved in the modulation of neural plasticity and neuron survival is the release of mBDNF and pro-BDNF in response to cellular membrane depolarization [59,60]. Indeed, the change in the electrical potential across the cell membrane occurring during cellular membrane depolarization leads to voltage-gated calcium channels opening, allowing the calcium influx into the cell and its increased intracellular concentration. This triggers the release of mBDNF and pro-BDNF [61,62]. Specifically, mBDNF is released through a process called exocytosis, in which vesicles containing mBDNF are transported to and fused with the plasma membrane, allowing the extracellular space release [36,63].

On the other hand, pro-BDNF is released through a process called ectodomain shedding, in which it is cleaved from the cell surface by a protein called protease. This process generates the mature form of the protein, the mBDNF, which can then be released in the extracellular compartment [36].

The ratio of pro-BDNF and mBDNF depends on brain regions and on specific stages of brain development: higher concentrations of pro-BDNF are observed in the early postnatal period; on the other hand, mBDNF is strongly expressed during adulthood [64]. Therefore, pro-BDNF can be considered a crucial factor modulating brain function by increasing neurogenesis and cell proliferation. It is well known that BDNF expression is regulated in an activity-dependent manner during neurodevelopment [65,66], and the mechanism of the activity-dependent production of BDNF-mRNA is due to the phosphorylation and the subsequent activation of the N-methyl-D-aspartate glutamate receptor (NMDAR). Concerning the pattern of expression of mBDNF in the adult brain, high levels of BDNF mRNA and protein have been discovered especially in hippocampus and cerebral cortex but also in the amygdala and in the cerebellum in both rodents and humans [67].

## 3. BDNF in the Brain: Its Role in Learning and Memory Processes

Several pieces of evidence support the crucial role of BDNF signalling in learning and memory processes both in physiological and pathological conditions [13,57,68,69].

The protective effects of BDNF have been extensively documented in the hippocampus, a brain region mainly involved in learning and memory functions [70,71,72,73,74]. Indeed, BDNF-TrkB signalling activates a cascade of intracellular events including downstream signalling pathways that promote functional and molecular underpinnings of learning and memory such as neurogenesis, synapse formation, and neuronal survival [32,75,76,77].

**Figure 1:**
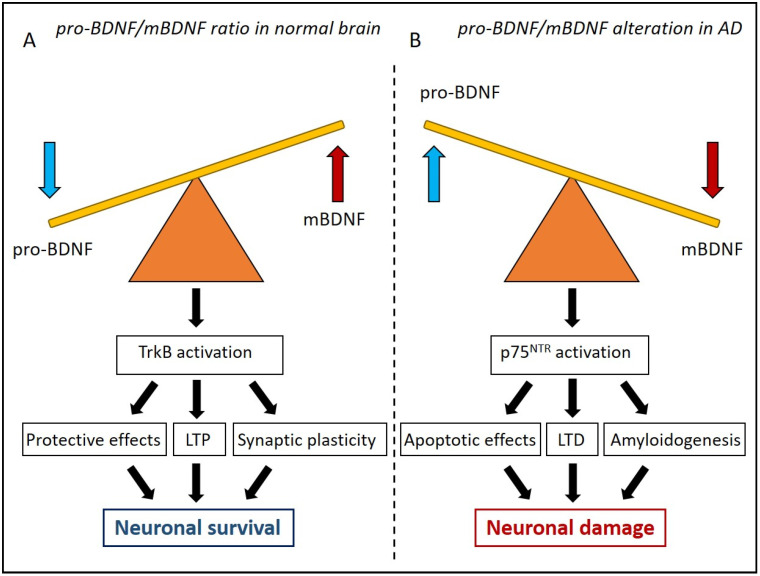
Schematic illustration of pro-BDNF/mBDNF ratio in normal brain and in Alzheimer’s disease (AD). (**A**) In a physiological condition, BDNF-TrkB signalling exerts protective effects, improving hippocampal long-term potentiation (LTP) and synaptic plasticity and promoting neuronal survival. (**B**) In AD, the alteration of pro-BDNF/mBDNF ratio and the accumulation of pro-BDNF leads to the activation of p75^NTR^ signalling pathway involved in programmed cell death, activation of long-term depression (LTD), and the biogenesis of Ab amyloid protein (amyloidogenesis), promoting neuronal damage.

In physiological conditions, mBDNF can decrease the activation of hippocampal GABA-ergic interneurons, thus improving LTP [9,78], the well-known major functional mechanism governing synaptic consolidation and memory formation [79,80,81] (Figure 1A).

Indeed, the synaptic plasticity refers to the underlying mechanisms for encoding and storing memories in the brain [82,83]. BDNF has been shown to be important for the formation and consolidation of long-term memories because of its release in response to neural activity and because its binding to receptors on the neuronal cellular membrane induces changes in the strength of neuronal connections [36]. Moreover, we know that changes in synaptic connections, together with an increase in the number, size, and complexity of dendritic spines, are the morphological basis of LTP and of memory storage [84,85]. Namely, BDNF-TrkB signalling can exert an effective role in promoting memory functions by favouring the expression of proteins regulating these plastic changes that underlie spatial and recognition memory processes [86,87,88,89,90]. Furthermore, BDNF has also been shown to be involved in the formation of recognition memories by promoting the growth and survival of neurons in the perirhinal cortex, a region of the brain that is important for object memory recognition [91,92,93].

Overall, for these reasons, understanding in detail BDNF’s role in the brain can be instrumental in developing new therapeutic strategies to counteract memory impairment in neurodegenerative diseases. Moreover, based on such considerations, identifying BDNF downregulation as an early molecular biomarker can be effective in disease prevention, as discussed below.

### Role of BDNF as an Early Biomarker in Neurodegenerative Disorders

Due to the pivotal role of BDNF in the learning and memory processes discussed above, it is not surprising that dysfunctions or downregulations of BDNF pathway are involved in the pathogenesis of neurodegenerative disorders including dementia, which are mainly characterized by neuronal damage, synaptic dysfunctions, and cognitive decline [94,95,96,97,98]. Indeed, evaluations of post-mortem brain tissues from patients with neurodegenerative diseases revealed that neuronal degeneration was strictly associated with a decrease in BDNF serum levels [99,100,101,102,103]. Accordingly, experimental evidence confirmed the association between BDNF downregulation and neuronal damage, showing a recovery of the neurodegenerative phenotype after BDNF administration and/or upregulation [104,105,106].

Considering the high relevance of the neurodegenerative diseases in our society and the lack of an effective therapeutic strategy, early prevention and diagnosis remain crucial instruments to counteract neurodegeneration. In this scenario, looking for early biomarkers of pathology can be an effective strategy. In this view, a biomarker can be very useful, considering that it is a qualitative and quantitative biological substance or characteristic that can define a normal or a pathological condition and may provide insights into the severity of the disease and the best therapeutic approach to be used [107,108,109].

Nowadays, thanks to the advances in technology, we can better detect, identify, and quantify biomarkers to validate them in clinical settings. BDNF is an emerging candidate among putative biomarkers, playing a crucial role in the diagnosis and treatment of neurodegeneration, as discussed below.

Thus, several approaches have been studied to evaluate the potential use of BDNF as biomarker in neurodegenerative diseases that require decision making in clinical practice. BDNF has been identified as a potential circulating biomarker for schizophrenia or depression and neurodegenerative diseases. However, even if many experimental studies confirm its role as a marker for the pathogenesis of several diseases, few reports describe methods to prove its role as a potential clinical prognostic indicator. One of these consists of measuring BDNF levels in various biological samples such as blood, cerebrospinal fluid, or post-mortem brain tissue. Modification of BDNF levels in serum has been described in epilepsy, psychiatric disorders, neurodegenerative diseases including Alzheimer’s disease, and schizophrenia [110]. Namely, low serum concentrations have also been shown in the cerebrovascular disease (CVD) as related to the metabolic syndrome and probably correlated to the oxidative stress unbalance, which is a pathogenetic marker of this condition, as well as demonstrated in other studies as a potential risk factor for the development of cerebrovascular disease [111]. Furthermore, a recent meta-analysis concluded that stroke severity is negatively correlated with BDNF levels. However, there is no correlation between BDNF levels and the volume of brain infarction or functional outcomes [112]. Thus, more studies are desirable to increase the evidence of the correlation between level of BDNF in the prognosis and outcome of diseases.

In vivo experimental studies have suggested that the altered stress-related responses might be related to BDNF deficiency as a common downstream mediator for environmental factors in the development of stress-related psychiatric profiles. Thus, BDNF genetic variation and coding polymorphisms have been recently studied, even if a multitude of variants have been recognized, and their functional role is still clinically unknown. However, to study genetic variations of BDNF or TrkB genes represents a new potential approach [113]. Evidence on a common polymorphism in the BDNF gene, known as *Val66Met*, has associated it with various psychiatric disorders, cognitive impairment, and changes in brain structure and function [114,115,116]. In spite of the promising results, more studies in both experimental and clinical models are needed in order to relate the genetic findings to the phenotypes of major related diseases. Interestingly, new insight on the role of BDNF Val66Met polymorphism seems to be promising for the role of the modulation of the stress susceptibility in the neuropsychiatric endophenotypes not only in the experimental models but also in humans [117].

It has been suggested that the Val66Met polymorphism may change the sensitivity to stress and plasticity modulating the action of the glucocorticoid stress hormones axis based on the hypothesis of the dichotomy that reduced activity of BDNF corresponds to decreased resilience and sensitivity to stress conditions [118]. Understanding the relationship of gene–environment interactions and differential activation of BDNF in the brain subjected to stress-related illnesses and maladaptive plasticity will be helpful for the development of novel diagnostic tools and treatments.

Among neurodegenerative disorders, AD is the most common cause of dementia [119,120,121]. AD is a progressive neurodegenerative disorder characterizes by a gradual loss of nerve cells and neural connections, leading to memory loss and cognitive decline [122,123,124]. Although there has been much important research in this field, no effective treatments have been found, mainly due to the incomplete knowledge about the molecular basis of this complex pathology. The pathological mechanism of AD involves the accumulation of beta-amyloid (Aβ) plaques and tau protein neurofibrillary tangles in the brain [125,126,127,128]. The accumulation and the spreading of these misfolding proteins are also associated with neuroinflammation and oxidative stress [129,130,131]. Indeed, it has been shown that increased neuroinflammatory markers and microglia activation increase Aβ plaques accumulation together with increased tau phosphorylation, favouring neurofibrillary tangles aggregation and causing synaptic damage and neuronal death [130,132,133]. Moreover, a core symptom in AD is memory impairment due to neuropathological changes in the limbic system [134] and specifically in the hippocampus, the brain region that is involved in learning and memory processes [77,135,136].

Indeed, in AD, the hippocampus is one of the first areas of the brain to be affected, and it is thought that the loss of BDNF in this brain region may contribute to the cognitive impairment observed in AD patients. A large amount of evidence has highlighted a decreased level of BDNF as well as mRNA related to BDNF signalling in the hippocampus of patients with severe AD [137,138] but also in other brain regions such as the entorhinal cortex, frontal cortex, temporal cortex, and parietal cortex [137,139,140,141,142,143,144,145,146]. Indeed, a decrease in BDNF protein and mRNA levels in cerebrospinal fluid (CSF) and peripheral blood in AD patients has been observed [100,147,148,149]. Therefore, it has been suggested that measuring BDNF levels in biological fluids can be considered promising tool for AD early diagnosis [7,148].

Considering the important role of BDNF in neuronal survival, its downregulation can severely compromise synaptic plasticity, the core pathological hallmark in AD. Indeed, transgenic mice with decreased levels of BDNF expression show consequent defects in LTP induction [150,151] (Figure 1B). On the other hand, high levels of BDNF were found in blood samples from AD patients, revealing conflicting results [152,153]. However, it is possible that the increase in blood BDNF concentration found in AD patients is due to a compensatory mechanism to fight early neurodegeneration or for immune cell activation. Shin and colleagues found an increase in BDNF serum levels in patients with mild cognitive impairment (MCI), a cognitive decline strongly associated to AD progression, whereas BDNF concentrations decreased in severe AD, supporting the hypothesis that BDNF was up-regulated only in an early preclinical stage of the pathology [153]. A hypothesis could be related to a failure of the trophic support that contributes to the neurodegenerative progression. One more explanation of these opposing results can be found in the complex and sophisticated regulation of pro-BDNF processing, considering the role of glial cells and plasmin inhibitors in the regulation of pro-BDNF modulation [60].

The alterations in the pro-BDNF/BDNF ratio in AD is more controversial. Interestingly, clinical evidence has demonstrated that pro-BDNF plays a central role in the pathogenesis of the disease [139,154] (Figure 1B). Michalski and Fahnestock found a decrease of about 40% in pro-BDNF protein levels in the parietal cortex of end-stage AD patients with respect to healthy subjects [146]. This significant pro-BDNF reduction is in line with the results obtained by Holsinger and collaborators, demonstrating a decrease in BDNF mRNA expression levels in the brain of AD patients [155].

The direct consequence of the accumulation of pro-BDNF is the activation of p75^NTR^ signalling pathway involved in both programmed cell death [156,157] and in the activation of the biogenesis of Aβ, which is known as amyloidogenesis [158] (Figure 1). Several research groups have confirmed these observations. Indeed, Gerenu and colleagues demonstrated in both a clinical and experimental model of AD that the soluble form of Aβ affects the proteolytic cleavage of pro-BDNF, suggesting that alterations in BDNF maturation can contribute to neuronal and cognitive dysfunctions [159] (Figure 1).

Chen and collaborators showed that the accumulation of pro-BDNF enhances the Aβ deposition, favouring senile plaques formation and thus accelerating learning and memory deficits in APPswePS1dE9 mice, a mouse model of AD [160]. Fleitas and co-workers also demonstrated that pro-BDNF play a role in mediating apoptosis in AD. Specifically, the authors studied the relative levels of mBDNF and pro-BDNF in AD patients, finding a significant increase in pro-BDNF in the hippocampus [161]. They also observed an increase in pro-BDNF levels in CSF of AD patients that was highly dependent on oxidative stress.

Overall, these results point out a relevant conclusion: both pro-BDNF/p75 signalling and the ratio pro-BDNF/BDNF measured in the CSF could be considered potential diagnostic biomarkers to develop new therapeutic approaches for AD. In addition, reducing pro-BDNF levels at a preclinical stage and targeting the conversion of pro-BDNF into mBDNF may contribute to AD prevention. For this reason, even if more studies are needed to determine the role of BDNF in the onset and progression of the neurodegenerative diseases, from a translational point of view, an interesting diagnostic strategy could be to monitor the levels of pro-BDNF/mBDNF during the evolution of AD to identify high-risk patients and to plan early therapeutic strategy.

Experimental and clinical studies have suggested that BDNF may serve as a biomarker also in neuropsychiatric disorders such as schizophrenia. Reduced levels of BDNF have been observed in the brain of people with schizophrenia, particularly in regions involved in cognitive processes, such as the prefrontal cortex and hippocampus [162,163]. Schizophrenia is associated with cognitive impairment including deficits in attention, memory, and executive function. Considering the role of BDNF in synaptic plasticity, which is crucial for cognitive functions, the reduced levels of BDNF in schizophrenia may contribute to the cognitive deficits observed. BDNF has been implicated in the mechanism of action of various antipsychotic medications used to treat schizophrenia. Studies have shown that the levels of BDNF increase in response to treatment with antipsychotics, particularly those that block dopamine receptors [164,165]. This suggests that BDNF may be a useful biomarker to monitor treatment response in schizophrenia.

Moreover, clinical studies have suggested that BDNF levels may also predict the course and outcome of schizophrenia. For example, reduced BDNF levels have been associated with a more severe form of the illness, poorer cognitive function, and a worse prognosis overall [166,167].

Therefore, the evidence so far suggests that BDNF abnormalities may contribute to the pathophysiology of this disorder and that measuring BDNF levels could be useful for diagnosis, treatment monitoring, and predicting the course of the illness.

It has been demonstrated that decreased levels of BDNF activity in the hippocampus are strictly related to depression. Indeed, studies have suggested that decreased BDNF levels can be associated with depression onset and progression [6,168]. Considering that depression is associated with a reduction in the size of neurons and/or neuronal death, neurotrophins represent good candidates to better understand the pathogenesis of depression and antidepressant drug actions. Indeed, experimental research in animal models has demonstrated that exposure to different types of stress can decrease BDNF expression, especially in hippocampus and prefrontal cortex [169,170], whereas antidepressant treatment enhances the expression of BDNF in the same brain regions [171,172].

## 4. BDNF in the Auditory System

BDNF plays a decisive role in the control of neuronal survival/death during neuronal development [64,173]. This is true not only for brain but also for the peripheral hearing organ, the cochlea [174,175,176].

The cochlea, which is in the inner ear, is characterized by several cell populations that are highly specialized and responsible for sound mechanoelectrical transduction. Hearing receptors include two major types of cells localized on the basilar membrane in the organ of Corti: the inner (IHCs) arranged in one row and the outer hair cells (OHCs) arranged in three rows. The hair cells play a key role in transducing sound-evoked mechanical motion into electrical signals; indeed, glutamatergic synapses between hair cells and the primary afferent fibres of the spiral ganglion neurons (SGNs) allow the up-spread of neuronal signals along the auditory pathway [131]. A great deal of research has extensively underlined the functional role of BDNF in the inner ear during neurodevelopment [11,177,178,179,180,181]. Several experimental studies in animal models have focused on the expression and functions of BDNF and its high-affinity receptor, TrkB, during the development of SGN innervation and regeneration [181,182,183].

Schimmang and collaborators demonstrated that TrkB mutant mice show a significant loss of SGNs during development [184]. Researchers have also shown that TrkB mRNA expression in the cochlea is developmentally regulated. For example, during embryonic development, TrkB mRNA expression is highest in SGNs, while in the adult cochlea, it is highest in the OHCs [185,186].

The role of BDNF in ear development has been supported by studies on mice carrying null mutations for BDNF and TrkB [187,188,189]. Indeed, studies on BDNF knock-out (^−/−^) mice revealed a synaptic damage with loss of sensory cell innervation, specifically in the apical cochlear turn [177,179]. Moreover, a specific loss of OHC/type II SGN synapses was found in both *BDNF*^−/−^ and *TrkB*^−/−^ mice [175,184,190,191]. In late postnatal stages, *BDNF*^−/−^ mice show a lack of afferent innervation in the apical region of the cochlea, while in older stages, the basal turn is affected [192]. These observations reveal a spatial gradient of BDNF expression along the cochlear spiral axis [185,193]. Indeed, BDNF is expressed during adulthood in a tonotopic gradient, with an increased expression toward high-frequency cochlear regions [181,194,195,196].

In adulthood, many studies concerning BDNF and its receptor expression in the inner ear have been reported. The expression of TrkB and the low-affinity receptor, p75^NTR^, has been found in SGNs in rodents [183,197]. Indeed, p75^NTR^ has been detected in human cochleae and localized in glial cells, central nerve fibres within the modiolus, and the osseous spiral lamina [198].

**Figure 2:**
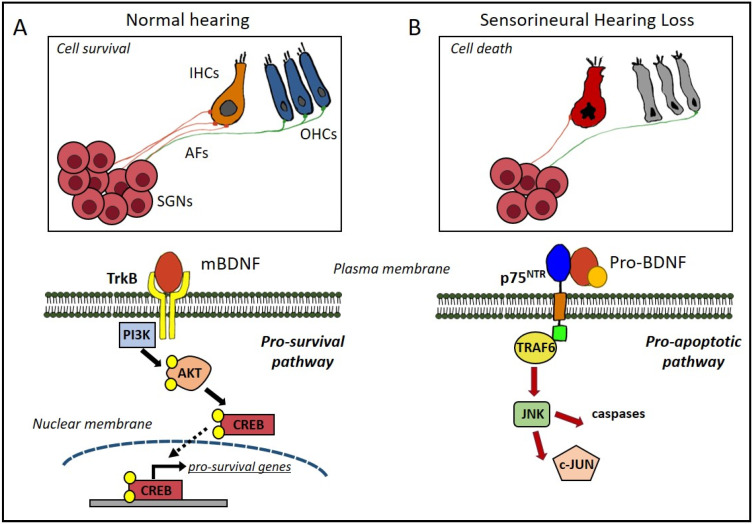
Schematic representation of TrkB and p75^NTR^ pathways in normal hearing and in sensorineural hearing loss. In normal hearing cochlea (**A**), mBDNF binds TrkB receptor on the plasma membrane of spiral ganglion neurons (SGNs) and activates PI3K/AKT pathway. PI3K phosphorylates and activates AKT (phosphorylation sites indicated by yellow spots), which in turn phosphorylates the transcription factor CREB, which translocates into the nucleus and induces pro-survival gene expression. In damaged cochlea (**B**), pro-BDNF binds the p75^NTR^ receptor on the SGN plasma membrane and activates signalling proteins such as TRAF6 and JNK, which are involved in the activation of c-JUN and caspases, leading to cell death. IHCs, inner hair cells; OHCs, outer hair cells; Afs, afferent fibres; SGNs, spiral ganglion neurons; TrkB, tropomyosin receptor kinase B; PI3K, phosphatidylinositide 3 kinase; AKT, protein kinase B; CREB, cAMP response element-binding protein; p75^NTR^, p75 neurotrophin receptor; TRAF6, TNF receptor-associated factor 6; JNK, c-Jun N-terminal kinases; c-JUN, transcription factor Jun.

Regarding BDNF expression pattern in cochlear structures during adulthood, although mRNA transcripts may be at very low levels [199,200], BDNF immunoreactivity has been observed in the organ of Corti [20,201]. Tan and Shepherd demonstrated that BDNF is expressed in the sensory and supporting cells in adult rats [20]. Interestingly, they observed that SGN degeneration in a model of aminoglycoside-induced hearing loss is related to a p75^NTR^-dependent activation of apoptosis together with alterations of the TrkB-downstream signalling promoting cell survival [20]. Wissel and co-workers demonstrated, by immunohistochemical studies of cochlear sections, a differential expression of BDNF and TrkB in SGNs in normal hearing and in rats with hearing loss treated with neomycin [197] (Figure 2).

Meltser and collaborators revealed differential expression pattern of pro-BDNF and TrkB after hearing loss induced by a temporary (TTS) or permanent (PTS) increase in the auditory thresholds caused by the exposure to a loud noise [202]. Only after PTS were a downregulation of TrkB expression and an upregulation of pro-BDNF found. This finding suggests that cell death related to PTS induces an activation of the pro-BDNF/p75^NTR^ pathway and a downregulation of the pro-survival BDNF/TrkB pathway, revealing distinct molecular mechanisms for different severity degrees of hearing loss.

Notably, a strongly modulation of BDNF expression has also been found in animal models of age-related hearing loss (ARHL) [203,204]. ARHL, i.e., presbycusis, refers to the increase in auditory thresholds occurring with advancing age, leading to hearing loss in the elderly [205,206,207]. Rüttiger and colleagues observed a significant reduction of BDNF levels, both at the RNA and protein level, in an animal model of presbycusis [203]. Interestingly, they showed a clear localization of BDNF protein within the central projections of the bipolar SGNs that was lost in aged hearing-impaired animals, suggesting a significant alteration in BDNF protein localization with advancing age.

These observations are in contrast with results obtained by Liu and collaborators in the human adult inner ear. The authors observed a strong expression of TrkB in neuronal somata and processes in the SGNs of human cochlear samples, even though BDNF immunostaining showed no positive results in the same samples [198]. In addition, the finding that BDNF in culture medium is necessary for human cochlear neuron survival supports its crucial role for cochlear neuronal viability [208].

### 4.1. Cochlear Damage: Sensorineural Hearing Loss

Hearing loss is a major public health concern and the fourth leading cause of disability [209]. It affects approximately 1/1000 new-borns and one-third of people over 65 years old and is disabling in ∼5% of the world population [210]. Clinically, hearing loss has been classified into three main types: conductive, sensorineural, and mixed hearing loss. Sensorineural hearing loss (SNHL) is the most common type and represents most of all hearing loss. SNHL refers to any cause of deafness due to a damage involving the cochlea, auditory nerve, or central nervous system. It is a heterogeneous disease with multiple aetiologies depending on the relationship between genetic and environmental factors including noise exposure, ototoxic drugs, and aging [211]. Remarkably, it is estimated that up to 60% of hearing loss is attributed to preventable causes [210]; however, diagnostic, prognostic, therapeutic, and pathognomonic biomarkers of the disease are lacking for the majority of ear pathologies. Although pure-tone audiometry provides relevant diagnostic information on the type, degree, and configuration of hearing loss, little differential diagnostic information can be obtained on auditory system dysfunction. Even if adjunctive information on the side of the lesion toward the auditory pathway from the inner ear to the brain can arise by the audiological battery including tympanometry, the acoustic stapedial reflex, otoacoustic emissions, electrophysiological tests, speech audiometry, and neuroimaging, few laboratory tests are helpful for the diagnostic definition of the pathology, and the precise diagnosis is unavailable for many SNHL aetiologies [212]. Increasing experimental findings support the evidence that neurodegeneration is a common pathophysiological mechanism underlying several SNHL such as ARHL, noise-induced hearing impairment, and drug ototoxicity. At the molecular level, these pathological conditions can be related to oxidative stress mechanisms, with increased reactive oxygen species (ROS) and decreased endogenous antioxidant defences leading to redox status unbalance in the cochlea [131,213,214]. Moreover, mitochondrial formation of ROS provides the upregulation of vascular biomarkers such as hypoxia-inducible factor α (HIF-1α) and the inflammatory response targeting synapses between IHCs and SGN terminals [211]. Thus, the interplay between oxidative stress and inflammation can play a crucial role in cochlear damage [213]. Furthermore, peripheral cochlear damage may trigger an up-spread dysfunction in the auditory pathway, leading to central mechanisms of maladaptive plasticity [215,216,217,218] as well as alterations in the balance of excitation/inhibition in neuronal networks of the auditory system [219,220,221,222,223]. Recently, we observed that noise exposure and early presbycusis causes morphological changes in neurons of the auditory cortex (ACx), such as decreased number of dendritic spine and altered dendritic complexity in pyramidal neurons of layer II/III of the ACx and in the hippocampus [207,224,225,226]. Moreover, several studies have demonstrated that SNHL can affect hippocampal functions by causing an alteration of neurotransmitter levels [227,228,229] and by decreasing neurogenesis [230,231]. Unfortunately, most molecular biomarkers that can be measured in blood are inflammatory markers or not limited to the inner ear and therefore are non-specific. On the other hand, the blood labyrinthine barrier (BLB) allows the maintenance of the inner ear fluid ionic homeostasis by its lower permeability that limits the flow of deleterious substances into the inner ear [232,233].

Because of the inconsistency of clinical biomarkers of SNHL, research in this field is challenging [234]. In this scenario, BDNF can exert a protective role, potentially contrasting cochlear molecular damage and preventing neuronal dysfunctions underlying hearing loss.

### 4.2. BDNF Protective Effects against Hearing Loss

SNHL is caused by irreversible damage with a substantial or complete loss of hair cells, resulting in permanent deafness [235]. A wide number of studies have shown that the loss of both OHCs and IHCs induces a subsequent degeneration of SGNs [236]. Hence, the administration of exogenous neurotrophic factors has been widely studied in animal models of SNHL, showing a pro-survival effect for auditory neurons in models of SNHL [176,237]. A wide number of studies have revealed that BDNF and NT-3 are the most common neurotrophic factors used in animal models of hearing loss [238,239,240,241,242,243,244,245,246,247,248,249,250,251,252,253].

The potential beneficial effect of BDNF has been widely tested in association with cochlear implants. Indeed, BDNF supplementation has been proposed for improving SGN survival and enhancing cochlear implant outcomes [254,255]. Both in vitro and in vivo studies showed that the exogenous application of BDNF improves not only SGN survival [241,256,257] but also potentiates the beneficial effects of an electrical stimulation of the auditory nerve [242,247,256,258,259,260].

In vitro studies [261,262] have shown the neuronal protective effect of BDNF against cochlear damage induced by ototoxic drugs such as aminoglycosides (i.e., gentamicin), therapeutic drugs (i.e., salicylates), and chemotherapeutic agents (i.e., cisplatin) [263,264,265]. These findings highlight a role of BDNF in the prevention of cochlear injury induced by ototoxicity, which accounts for one of the main causes of SNHL.

Additionally, BDNF has been shown to promote neuronal survival of afferent cochlear neurons in several animal models of hearing loss [193,244,250,266].

One of the major challenges for in vivo studies is the administration root of protective molecules, which can determine their effectiveness. Direct intracochlear administration of neurotrophins using an osmotic pump has been demonstrated to be more effective than the delivery in the round window in terms of SGN preservation [267]. However, this method is more invasive, increasing the risk of infection and loss of residual hearing. Nevertheless, the limitation of this delivery system can be overcome by combining intracochlear administration of neurotrophins with cochlear implant surgery [268].

Less-invasive techniques include intratympanic delivery [269] and the use of specific biomaterials such as hydrogel [270,271,272], gelfoam [250], and alginate beads [239], preventing the rapid flowing of the molecules through the Eustachian tube.

A novel, promising therapeutic formulation of BDNF known as OTO-413 has been recently developed by the biopharmaceutical company Otonomy. OTO-413 is a formulation of human recombinant BDNF combined with a thermos-reversible polymer called poloxamer P407, allowing drug delivery for several weeks from a single intra-tympanic (IT) injection [273], thus ensuring a long-lasting protective effect. Indeed, the effects of OTO-413 on hearing loss have been studied in preclinical models, and the results have been promising. In animal studies, OTO-413 has been shown to promote the growth and regeneration of hair cells in the inner ear, ameliorating hearing function [273,274]. Piu and collaborators demonstrated that a single IT injection of OTO-413 has a beneficial effect in re-establishing synaptic connections between SGNs and hair cells in a rat model of cochlear synaptopathy induced by noise exposure [274].

Clinical trials of OTO-413 are currently in a phase 1/2 study for the treatment of speech-in-noise hearing impairment, and the drug is under evaluation to test its safety and efficacy in human subjects with hearing loss, and at the moment, no significant adverse effects have been observed.

Finally, among possible therapeutic strategies, cell-based therapy represents a promising approach allowing a permanent neurotrophin delivery into the cochlea. Rejali and co-workers used a modified cochlear electrode coated with allogeneic fibroblasts expressing and secreting BDNF to obtain SGN survival [245]. The protective effect of the BDNF-secreting electrode was evident in the basal turn and less significant in the apical cochlear region, reflecting the spatial gradient of BDNF diffusion in the *scala tympani*, where the implant is positioned. Warnecke and collaborators also demonstrated that the electrode coated with cells secreting BDNF enhances SGN survival [275]. The effectiveness of cell-based drug delivery strategies can also overcome the potential toxicity and infection risk of viral vectors for gene-therapy [276,277].

The strength of cell-based techniques is the continuous supply of neurotrophic factors to SGNs, rendering this delivery system a good candidate for clinical application, even if the assessment of the long-term effects (such as risk of tumorigenesis) of elevated neurotrophin concentration needs further investigations.

## 5. The Role of BDNF in Olfaction

The BDNF gene expression is high in various parts of the central nervous system involved in olfaction, including the hippocampus and olfactory bulb [278].

The expression of neurotrophins in the olfactory epithelium suggests that they may play a role in the maintenance and repair of the olfactory system in adults.

Nibu and co-workers investigated the expression of TrkB in the olfactory epithelium of mice during development and aging and demonstrated that TrkB is expressed in the olfactory epithelium throughout development, with the expression levels peaking around postnatal day 3–5 and gradually decreasing thereafter. In aged mice, the expression of TrkB was reduced compared to young adult mice [279]. This study suggests that TrkB plays important roles in the development of the olfactory epithelium and that its expression levels may decline with age.

Feron and collaborators analysed the expression of four neurotrophins (NGF, BDNF, NT-3, and NT-4) and their receptors in the olfactory epithelium of adult humans. They demonstrated that all four neurotrophins and their receptors were expressed in the olfactory epithelium and that the expression of these neurotrophins varied across different cell types in the olfactory epithelium. For example, NGF was primarily expressed in the supporting cells, while BDNF was mainly expressed in the olfactory sensory neurons [280].

Numerous studies have demonstrated the high regenerative capacity of neurons of the olfactory system (OS), specifically of the olfactory epithelium, providing an interesting model to study the molecular bases of both neurogenesis and neuronal differentiation. For instance, the OS exhibits extraordinary neural plasticity, whose cognitive and cellular mechanisms, although extensively studied, remain under investigation. Understanding the neural basis of plasticity in the OS may provide insight into the brain mechanisms underlying recovery and reorganization of olfaction [281].

Olfactory loss is among the first symptoms of neurodegenerative disorders. The mechanism of olfactory disturbance in neurodegenerative diseases involves neurotoxic effects, which include atrophy of hippocampal cells due to abnormally low concentrations of neurotrophic factors, including BDNF. Further, BDNF can promote dopaminergic neuron differentiation [281].

Accordingly, BDNF is a well-known regulator of the dopamine system and is described as candidate for treating Parkinson’s disease due to its dual neuroprotective and neurogenic properties. However, it is unclear whether increased dopamine innervation occurs developmentally or due to increased adult neurogenesis of dopamine neurons, which is an intriguing hypothesis remaining a subject of intensive debate [282].

Thus far, the neurogenetic basis of variability in human olfactory function remains elusive, but BDNF, the most abundant neurotrophin in the brain, plays an important role in cognitive function and memory as well as in olfaction [283,284].

BDNF seems to be a key element for adaptive modulation of the OS at both cellular and behavioural levels [283]. According to recent literature, BDNF may facilitate neurogenesis of the olfactory bulb and neuroepithelium, whereas the downregulation of BDNF is associated with apoptosis in the olfactory bulb. In mice, impaired BDNF signalling could impact the ability to distinguish odours. Interestingly, when olfactory training is conducted, BDNF increases; thus, upregulation of BDNF mRNA in the neuroepithelium is associated with the restoration of olfactory function after olfactory training [281].

Furthermore, the presence of the BDNF polymorphism Val66Met (rs6265), the variant of the Met allele, can enhance adaptive neural reorganization to improve olfactory ability. Indeed, it is associated with improved olfactory identification and further engagement of the semantic memory system within the olfactory network in an allelic dosage-dependent manner [283].

The BDNF Met allele is also associated with preserved olfactory function and increased risk of “false alarms” when no odours are present. In addition to BDNF Met allele, phantosmia is mainly associated with female gender, vascular risk factors, and parosmia. However, it is of interest to note that variables frequently considered to be beneficial for olfactory function, such as female gender and BDNF met allele, are also associated with the experience of phantosmia [285].

Hedner et al. reported that carriers of the BDNF Val allele exhibited a higher age-related olfactory impairment than Met allele carriers, perhaps due to the gene’s role in neural plasticity [286].

In addition, Biju and collaborators, in a study on mice, suggested that the presence of primarily pro-BDNF in the olfactory bulb has a neurotrophic role in the OS. Thus, pro-BDNF might protect olfactory mitral cells from the effects of apoptotic changes induced by odour sensory deprivation [287].

Previous results have also shown that BDNF is involved in neonatal rats’ olfactory association learning. Further studies have also proven a close relationship between BDNF and mitochondria. In fact, BDNF might improve the respiratory efficiency of brain mitochondria, and oxidative stress might induce dysfunction in mitochondria, reducing the level of BDNF [288].

Lateral olfactory tract usher substance (LOTUS) contributes to axonal tract formation in the developing brain and axonal regeneration in the adult brain. According to recent studies, BDNF increases the expression of LOTUS in cultured neurons through the TrkB signalling pathway. BDNF may act as a positive regulator of LOTUS expression, suggesting that BDNF might synergistically affect axon regrowth through the upregulation of LOTUS expression. Furthermore, these findings provide new insight into the notion that axon growth-promoting effects of BDNF include an indirect action of LOTUS and that BDNF acts as a positive regulator of LOTUS expression. Eventually, the increased LOTUS expression induced by BDNF may represent a novel therapeutic strategy for neuronal regeneration after CNS injury [289].

BDNF is widely expressed in all types of brain cells, especially M2 microglia. There are two phenotypes for microglia: the classic phenotype M1 and the alternative phenotype M2. M1 microglia aggravate brain injury and impede the repair of the CNS by producing pro-inflammatory factors (IL-6 and IL1-β), whereas M2 microglia promotes the repair of neural damage and the survival of brain cells by releasing anti-inflammatory factors (IL-10 and IL-4) and some neurotrophic factors including BDNF. This factor promotes neuronal growth, reduces the loss of neurons, and promotes neurogenesis as well, thus highlighting the importance of BDNF in the repair of neurological damage [290].

## 6. Conclusions

Taken together, clinical and experimental evidence highlight the possibility to use the neurotrophic factor BDNF as a key molecule to counteract neuronal damage and for its neuroprotective effects in both cognitive and sensory neurodegeneration. Several studies have confirmed the association between decreased levels of BDNF expression and neuronal damage, showing a recovery of the neurodegenerative phenotype after BDNF upregulation. Indeed, studies have demonstrated that exogenous administration of BDNF may be considered a promising tool for diverse types of neurodegenerative disorders including brain disease, olfactory impairment, and sensorineural hearing loss.

## Data Availability

Not applicable.
